# Supplementary material for: The Evolution and Characterization of the RNA Interference Pathways in Lophotrochozoa
Source: Genome Biol Evol. 2024 May 7;16(5):evae098. doi: 10.1093/gbe/evae098 (PMC11114477; doi:10.1093/gbe/evae098)
Supplement: evae098_Supplementary_Data [file evae098_supplementary_data.zip › Supplementary_materials.zip!/selected_miRNAs/CrGi_selected_miRNAs/CrGi_NC_047563.1_18056.pdf]

Provisional ID : NC_047563.1_18056
Score total : 144.9
Score for star read(s) : 3.9
Score for read counts : 138.3
Score for mfe : 1.8
Score for randfold : 1.6
Score for cons. seed : -0.6
Total read count : 283
Mature read count : 247
Loop read count : 0
Star read count : 36

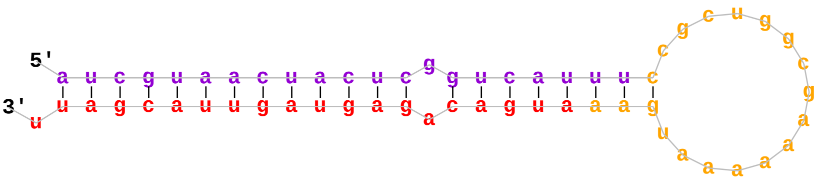

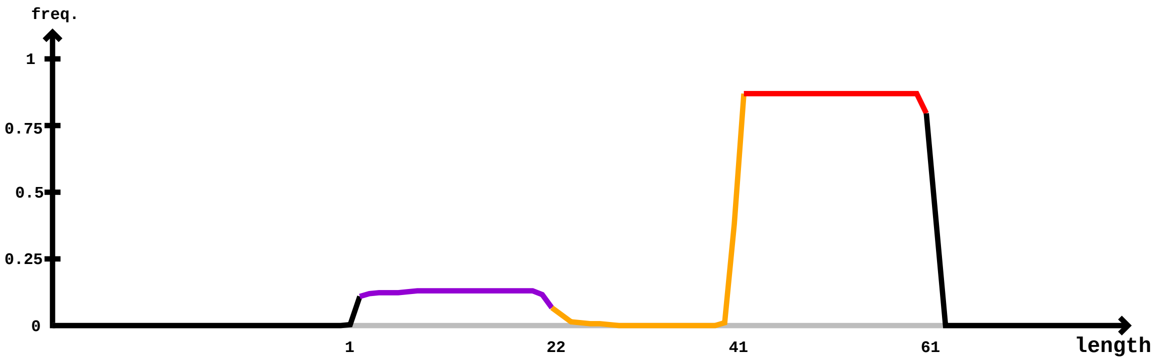


```
                                   Star                                  Mature
5'- gaggccuuccgagugcacuauuuacaugcaaucguaacuacucggucauuuccgcuggcgaaaaaaugaaaugacagaguaguuacgauuguauuuacauguagaucagc   -3'    obs
    gaggccuuccgagugcacuauuuacaugcaaucguaacuacucggucauuuccgcuggcgaaaaaaugaaaugacagaguaguuacgauuguauuuacauguagaucagc          exp
    .((((((....)).)).))((((((((((((((((((((((((.((((((((...............)))))))).)))))))))))))))))......))))))))).... reads   mm    sample
    ..............................aucguaacuacucggucau.............................................................  2       0     Ov2
    ..............................aucguaacuacucggucauu............................................................  2       0     Ov2
    ..............................aucguaacuacucggucauuu...........................................................  2       0     Ov2
    ....................................acuacucggucauuuccgcuC.....................................................  1       1     Ov2
    ....................................................................aaaugacagaguaguuacgau.....................  1       0     Ov2
    ....................................................................aaaugGcagaguaguuacgauug...................  1       1     Ov2
    .....................................................................aaugacagaguaguuacUauu....................  1       1     Ov2
    .....................................................................aaugacagaguaguuacgauu....................  15      0     Ov2
    ......................................................................augacagaguaguuacgau.....................  3       0     Ov2
    ......................................................................augacagaguaguuacgauu....................  25      0     Ov2

    ..............................aucguaacuacucggucau.............................................................  1       0     Ov1
    ..............................aucguaacuacucggucauu............................................................  4       0     Ov1
    ..............................aucguaacuacucggucauuu...........................................................  4       0     Ov1
    ...............................ucguaacuacucggucauuuc..........................................................  1       0     Ov1
    ................................cguaacuacucggCcauuuccg........................................................  1       1     Ov1
    ...................................aacuacucggCcauuuccgcu......................................................  1       1     Ov1
    ....................................................................aaaugacagaguaguuacgau.....................  1       0     Ov1
    .....................................................................aaugacagaguaguuacgau.....................  4       0     Ov1
    .....................................................................aaugacagaguaguuacUauu....................  1       1     Ov1
    .....................................................................aaugacagaguaguuacgauu....................  15      0     Ov1
    ......................................................................augacagaguaguuacgau.....................  1       0     Ov1
    ......................................................................augacagaguaguuacgauu....................  23      0     Ov1

    ..............................aucguaacuacucggucau.............................................................  1       0     Mt1
    ..............................aucguaacuacucggucauu............................................................  2       0     Mt1
    ..............................aucguaacuacucggucauuu...........................................................  1       0     Mt1
    ...............................ucguaacuacucggCcauuucc.........................................................  1       1     Mt1
    .....................................................................aaugacagaguaguuacgauu....................  32      0     Mt1
    ......................................................................augacagaguaguuacgau.....................  4       0     Mt1
    ......................................................................augacagaguaguuacgauu....................  35      0     Mt1
    ......................................................................augacagaguaguuacgauug...................  1       0     Mt1

    .............................aaucguaacuacucUgucauu............................................................  1       1     Mt2
```

Star Mature

```
gaggccuuccgagugcacuauuuacaugcaaucguaacuacucggucauuuccgcuggcgaaaaaaugaaaugacagaguaguuacgauuguauuuacauguagaucagc
..............................aucguaacuacucggucauu............................................................ 5 0 Mt2
..............................aucguaacuacucggucauuu........................................................... 6 0 Mt2
...............................ucguaacuacucggucauuu........................................................... 1 0 Mt2
.....................................................................aaugacagaguaguuacgau..................... 4 0 Mt2
.....................................................................aaugacagaguaguuacUau..................... 1 1 Mt2
.....................................................................aaugacagaguaguuacgauu.................... 31 0 Mt2
.....................................................................aaugacagaguaguuacgauug................... 1 0 Mt2
......................................................................augacagaguaguuacgau..................... 2 0 Mt2
......................................................................augacagaguaguuacgauu.................... 44 0 Mt2
......................................................................augacagaguaguuacgauug................... 1 0 Mt2
```
